# The Causation of Anemia and the Blood-Changes Produced by Uric Acid

**Published:** 1893-10

**Authors:** 


### THE CAUSATION OF ANEMIA AND THE BLOOD-CHANGES PRODUCED BY URIC ACID.

Dr. Alexander Haig read a paper on this subject at the recent meeting of the British Medical Association. He pointed out that his previous researches on "anemic attacks," paroxysmal hemoglobinuria, splenic leucocythemia, chlorosis, and anemia, showed that these troubles are very commonly associated with excess of uric acid in the blood and urine. The excess of uric acid is not the result of the blood-changes, but their cause; for the uric acid fluctuation begins before there are any blood-changes. The speaker said that he had the power, by influencing the solubility of uric acid, to bring it in excess through the blood into the urine at pleasure, and it is also possible to increase the uric acid in the blood and urine by taking a known quantity by the mouth. Applying this knowledge to the experimental production of blood-changes, he said the value of the blood fraction $\frac{\text{Per cent. Hemoglobin}}{\text{Per cent. Red cells}}$ varies with the amount of the uric acid passing through the blood from day to day; and by intentionally increasing the amount of uric acid, definite and distinct falls in the value of the blood fraction can be produced. Uric acid is more powerful than iron, and when in excess in the blood, will prevent iron from raising the value of the blood fraction; hence the observation of Murchison, that iron will not act when the liver is out of order, for dyspepsia entails an excess of uric acid in the blood. Others have produced similar blood-changes by the administration of thyroid extracts, which are rich in extractives closely related to uric acid; and a patient under this treatment gave undoubted clinical signs of uric acidemia. The author explained, by means of this uric acid causation, the anemia and chlorosis in girls, and the relation of these troubles to the function of menstruation; also the similar effect of fevers, tropical climates, and diet. As a result of these observations, he drew the conclusion, that anemia can be prevented by taking care not to introduce an excess of uric acid into the body, and can be cured by clearing it out of the blood.—*Medical Record.*

